# Case Report: QT Prolongation and Abortive Sudden Death Observed in an 85-Year-Old Female Patient With Advanced Lung Cancer Treated With Tyrosine Kinase Inhibitor Osimertinib

**DOI:** 10.3389/fcvm.2021.655808

**Published:** 2021-03-19

**Authors:** Moë Kondo, Megumi Kisanuki, Yosuke Kokawa, Seiichiro Gohara, Osamu Kawano, Shuntaro Kagiyama, Toru Maruyama, Keita Odashiro, Yoshihiko Maehara

**Affiliations:** ^1^Division of Cardiology, Department of Internal Medicine, Kyushu Central Hospital, Fukuoka, Japan; ^2^Department of Hematology, Oncology and Cardiovascular Medicine, Kyushu University Hospital, Fukuoka, Japan

**Keywords:** cancer, herbal drug, osimertinib, QT prolongation, cardiooncology

## Abstract

Cardiac arrest occurred in an 85-year-old female administered osimertinib for advanced lung cancer expressing epidermal growth factor receptor (EGFR) mutations. Electrocardiogram (ECG) recorded at recurrence of spontaneous circulation showed sinus rhythm associated with mild QT prolongation (QTc = 455 ms) to which silent myocardial ischemia and coadministration of itraconazole and herbal drug causing hypokalemia (2.1 mEq/L) may have contributed. Discontinuation of osimertinib, itraconazole and herbal drug, potassium supplementation and percutaneous coronary intervention alleviated QT prolongation (QTc = 432 ms). Osimertinib is the third-generation tyrosine kinase inhibitor lengthening QT interval, and careful monitoring of ECG, serum potassium and drugs coadministered during chemotherapy including osimertinib are highly required.

## Introduction

Cardiovascular complications of anticancer drugs are the main interest within the field of cardiooncology (1). Osimertinib, a third-generation tyrosine kinase inhibitor (TKI), is a first-line therapy for patients with cancer expressing epidermal growth factor receptor (EGFR) mutations. This anticancer drug has adverse effects including cardiotoxicity such as coronary tonus elevation and QT prolongation in electrocardiogram (ECG), because vascular and cardiac potassium channels are regulated largely by EGFR tyrosine kinase (2). However, osimertinib-induced QT prolongation leading to the possible development of Torsades de Pointes (TdP) has received less attention (3). We herein describe a case of 85-year-old female patient with EGFR driver mutant lung cancer treated with osimertinib, which lengthened QT interval causing abortive sudden cardiac death (SCD).

## Case Description

An 85-year-old female was conveyed by ambulance to the emergency room of our hospital due to cardiac arrest. She had lost her consciousness at 15:15 after she visited the outpatient clinic of another hospital for the treatment with advanced lung cancer (adenocarcinoma: cT2aN1M1b, Stage IVB). Bystander resuscitation was started immediately after the cardiac arrest, and emergency call was requested at 15:18. Ambulance crews confirmed cardiac arrest at 15:28, and she restored spontaneous circulation at 15:34 after the first discharge of public automated external defibrillator (AED). When she admitted to our hospital by ambulance at 15:40, her body temperature was 36.2°C, consciousness level in Glasgow Coma Scale was E1V1M1, and reflex to light was prompt. Blood pressure was 154–103 mmHg, pulse was regular, and its rate was 81 bpm. No abnormal physical findings were found. She had been started administration of oral osimertinib (40 mg once daily) as a first-line therapy of lung cancer after confirming EGFR mutation (T790M), oral itraconazole (200 mg once daily) for prevention of opportunistic fungal infection and crude herbal anticancer drug used in Chinese traditional medicine (TJ-48). She had no complaint of chest oppression or syncope nor family history of unexpected or abortive SCD in her relatives.

**Figure 1:**
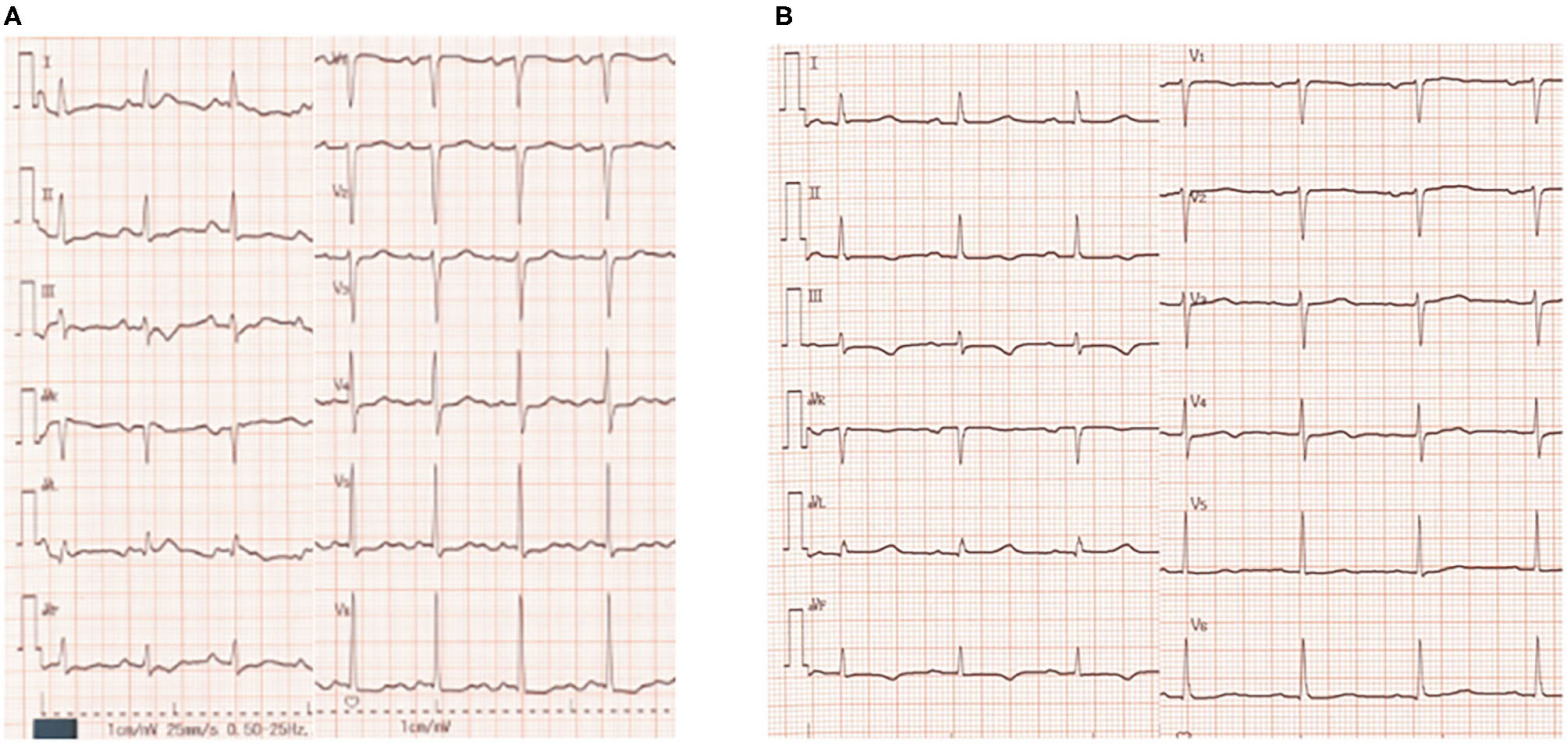
Twelve-lead electrocardiogram (ECG) on admission **(A)** and before discharge **(B)**. QTc interval was 455 ms **(A)** and 432 ms **(B)**, respectively. Herat rate correction of QT interval was performed by Bazett's formula.

**Figure 2:**
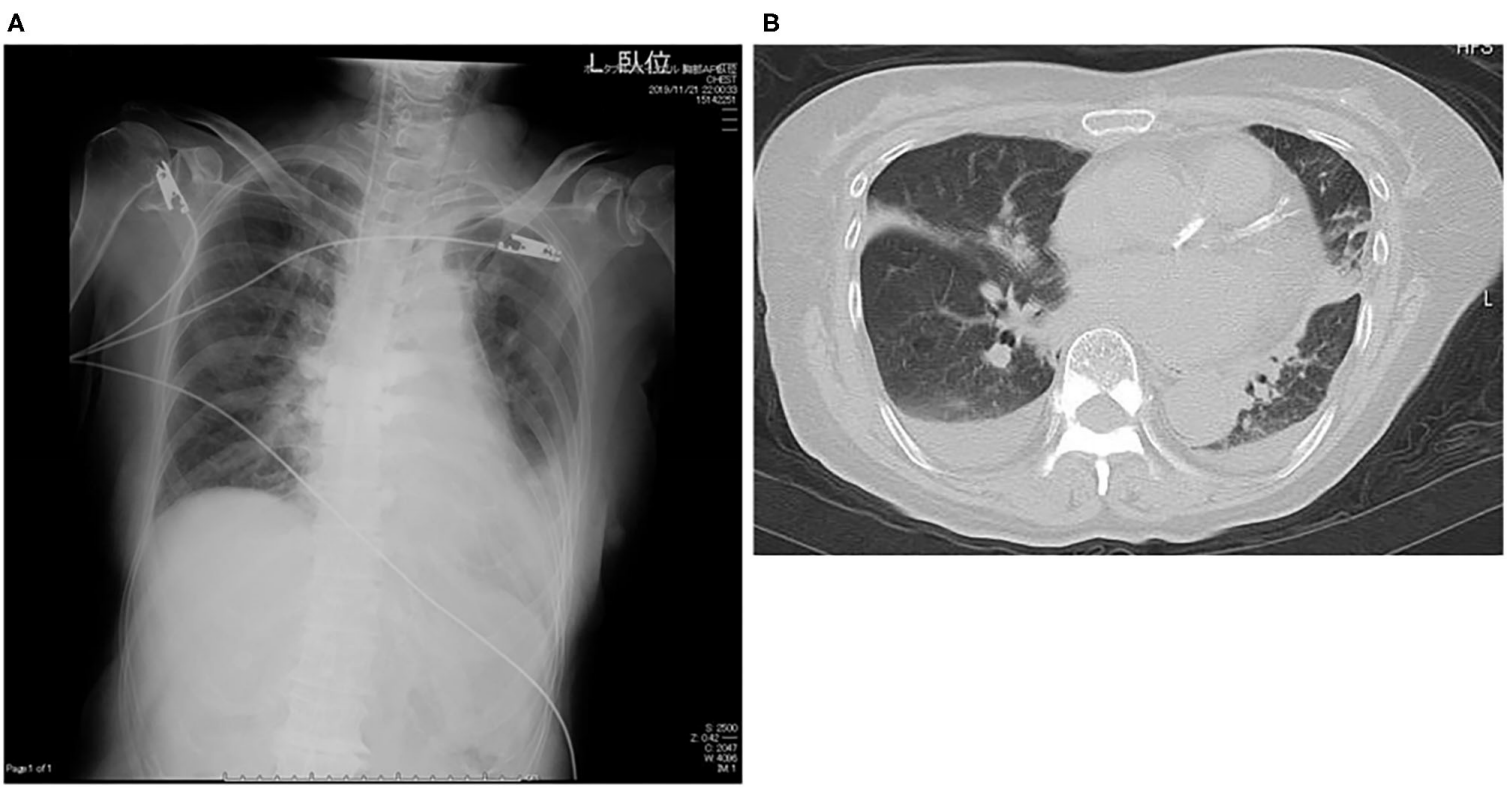
Chest X-ray on admission **(A)** and chest computed tomography during hospitalization **(B)**.

**Figure 3:**
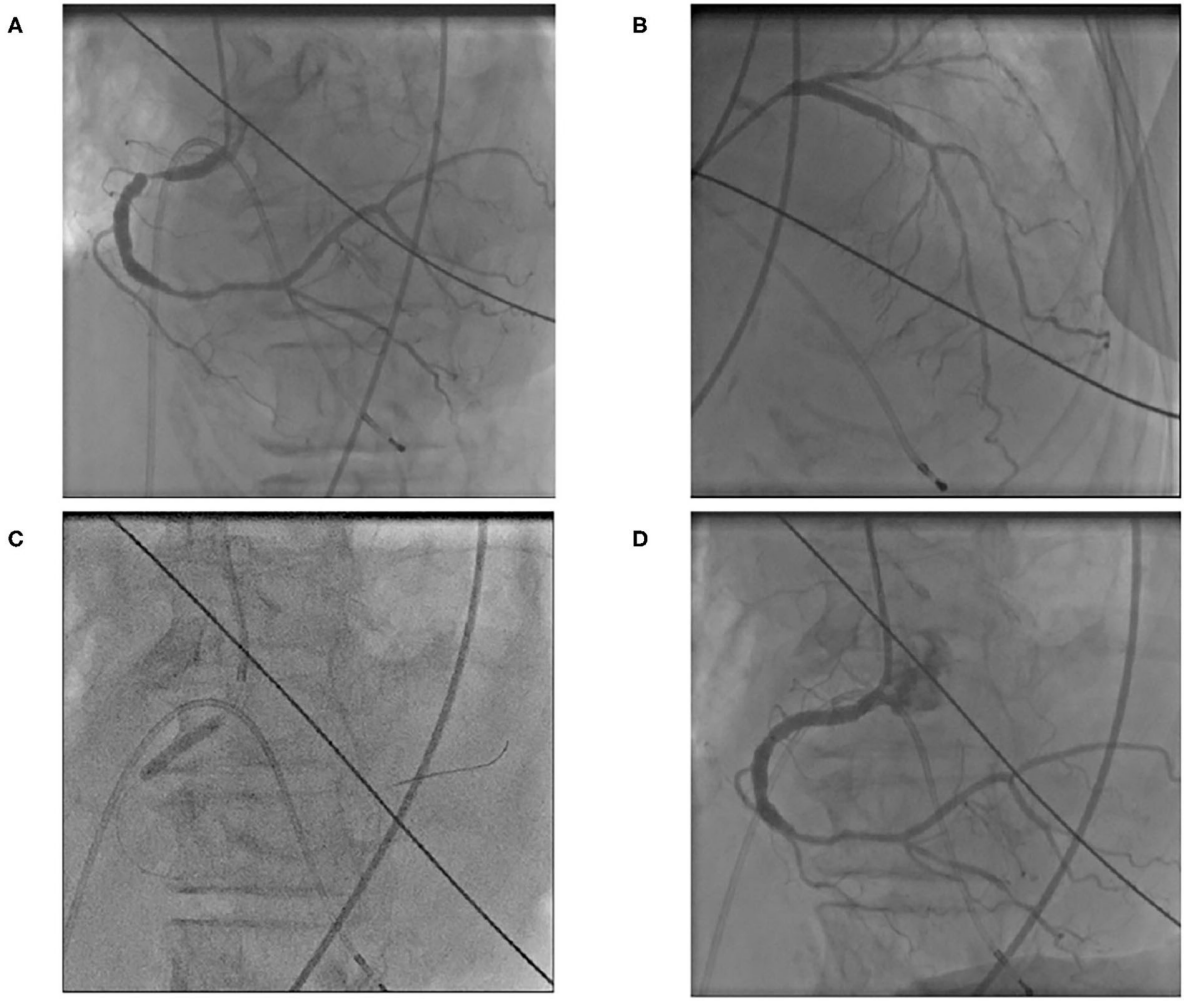
Right coronary angiogram showed severe (99%) stenotic lesion in segment 1 **(A)**. Collateral circulation from left anterior descending artery was visualized **(B)**. Stent implantation was performed **(C)** and right coronary angioplasty was completed **(D)**.

Laboratory examination on admission included serum chemistry showing total protein 5.8 g/dL, albumin 3.2 g/dL, CK 215 IU/L (CK-MB 19 IU/L), CRP 0.3 mg/dL, BNP 348.5 pg/mL, troponin T (TnT) 0.01 ng/mL, Na 139 mEq/L, Ca 9.2 mEq/L, and K 2.1 mEq/L. Hematology indicated mild anemia (hemoglobin of 10.3 g/dl) associated with white blood cell and platelet counts of 8,500/μL and 25.1 × 10^4^/μL, respectively. ECG showed sinus rhythm, left axis deviation, flat or inverted T waves observed in II, III, aV_F_, V_5−6_, and QT prolongation (grade I), i.e., QT interval corrected by heart rate (QTc = QT/RR^0.5^) was 455 ms (Figure 1A). Chest X-ray showed cardiomegaly and chest computed tomography demonstrated bilateral pleural effusion (Figures 2A,B). Ejection fraction was 64% on echocardiogram. Chemotherapeutic regimen including osimertinib, oral itraconazole and herbal medicine (TJ-48) were discontinued, and supplementation of potassium was started. Considering possible ischemic T wave changes (Figure 1A), coronary angiogram was performed. Stenotic lesion (99%) was found in the right coronary artery (segment 1) and collateral circulation supplied from left anterior descending artery was observed (Figures 3A,B). Percutaneous coronary intervention (PCI) was completed successfully by stent implantation at the culprit lesion (Figures 3C,D). QTc interval after the successful PCI and restoration of hypokalemia (K of 4.0 mEq/L) was 432 ms (Figure 1B), and she discharged on foot 33 days after admission without any neurological sequelae of cardiac arrest. Administration of osimertinib (40 mg once daily) was resumed in the hospital where she had been started chemotherapy under the monitoring of ECG and serum potassium concentration while coadministration of itraconazole and herbal drug was interrupted.

## Discussion

Osimertinib is an oral, third-generation TKI as an emerging therapeutic modality in various cancers with EGFR T790M mutation (2). One of the obstacles for wide use of this promising anticancer agent is QT prolongation. QT interval is lengthened as a result of ventricular potassium current reduction and late sodium current augmentation (4). Bian et al. (5) reported a first case of 85-year-old male patient with advanced non-small cell lung cancer associated with TdP induced by oral osimertinib (80 mg once daily) and concomitant intravenous moxifloxacin, a broad-spectrum fluoroquinolone antibiotics known to lengthen the QT interval (QTc of 484 ms). Hypokalemia (2.94 mEq/L), impaired left ventricular ejection fraction (41%) and coadministration of moxifloxacin were concluded to underlie the development of TdP. They lost this case and hence cautioned cardio-oncologists to identify cancer patients at high risk of QT prolongation leading to the development of TdP (5). Cancer development and apoptosis is governed by driver mutation of signal transduction process mediated by several growth factor receptor including EGFR which is linked to many potassium channels, i.e., Kv1.3 (6), Kv10.1 (7), Kv4.3 (8), and Kir2.3 (9) are modulated by EGFR kinase via phosphorylation of tyrosine in their specific residues. These potassium channels regulate vascular tonus and QT interval in ECG. Therefore, EGFR-TKI shows variable cardiotoxicity including vascular constriction and QT prolongation. Although the serum concentration of osimertinib was not monitored, and genetic testing of hereditary long QT syndrome was not performed in this case, careful ECG monitoring is essential for cancer patients under the chemotherapy including osimertinib (10).

QT interval is regulated strictly by many factors including basic heart rate, electrolytes, body temperature, sex hormones, and so on. Therefore, key factor of QT prolongation in this case is not determined easily. This case is an 85-year-old female patient, and old woman *per se* is a risk factor of QT prolongation (1). Oral itraconazole, a representative antifungal agent, is known to elevate the serum concentration of osimertinib due to competitive metabolism mediated by cytochrome P450 3A4 (CYP3A4). Chronic myocardial ischemia requiring PCI and hypokalemia (2.1 mEq/L) caused partly by Chinese herbal drug may have also contributed to the QT prolongation. Osimertinib causes coronary tonus elevation induced by vascular constriction mediated by inhibition of vascular EGFR (10), which may have aggravated silent myocardial ischemia in this case. Chinese traditional medicine of TJ-48 is used widely in Japan under the adjuvant anticancer therapeutic strategy as in this case. This herbal medicine is reported to enhance the efficacy of chemotherapy and to attenuate the adverse effects and complications of anticancer treatment (11). However, long-term use of Chinese herbal medicines is sometimes associated with hypokalemia caused by pseudoaldosteronism that is evoked by licorice contained in many herbal medicines. Although the incidence of hypokalemia induced by the administration of TJ-48 is not reported, such incidence by other Chinese medicine ranges from 6.6% (TJ-54) to 33% (TJ-9 and TJ-68) according to the Japanese Adverse Drug Report Database (12).

Considering the serum chemistry (normal CK-MB isozyme and negative TnT test) and echocardiographic left ventricular wall motion, cardiac arrest caused by acute coronary syndrome was unlikely. Silent myocardial ischemia is associated with QT prolongation caused by electrical instability and sympathovagal imbalance (13), and QT prolongation *per se* is responsible for SCD whatever the causes to prolong QT intervals are (14). Well-developed collateral circulation may have made coronary artery disease silent in this case (Figures 3A,B). This case description includes limitations, i.e., ECG was not retrieved from AED, serum TnT was assayed only once, and serum concentration of osimertinib was not assayed. In spite of these limitations, cardiac arrest in this case was supposed to be due to possible development of TdP based on several predisposing factors lengthening QT interval. Because defibrillation by means of public AED restored her spontaneous circulation, and recurrence of cardiac arrest was prevented after normalization of QT interval by elective PCI, discontinuation of causative drugs and potassium supplementation. Although QT prolongation in this case was grade I, this interval was measured at rest and supine position. QT interval dynamicity is accentuated by daily physical activity (15), and the relation between sudden increase in QT interval and the development of TdP is open to debate. This case taught us the necessity of collaboration between oncologists and cardiologists in the earlier clinical course of cancer patients.

## Conclusions

We have described an 85-year-old female patient with EGFR-mutant advanced lung cancer treated with standard regimen of osimertinib, which underlay in part grade I QT prolongation causing abortive SCD. Reversible factors promoting QT prolongation were removed after admission and this patient was discharged safely. Although osimertinib-induced QT prolongation is mild to moderate (grade I or II) and not frequent (3–10%) in AURA study and FLAURA trial to verify the safety and efficacy of osimertinib (16, 17), TdP may be induced by standard dose of osimertinib, when multiple risk factors to lengthen QT interval are incidentally overlapped. Careful ECG monitoring and appropriate management of risk factors lengthening QT interval such as interacting drugs, electrolyte imbalance and cardiac ischemia during chemotherapy including osimertinib are highly required.

## Data Availability Statement

The raw data supporting the conclusions of this article will be made available by the authors, without undue reservation.

## Ethics Statement

Written informed consent was obtained from the individual(s) for the publication of any potentially identifiable images or data included in this article.

## Author Contributions

This case report article was carried out in collaboration between all coauthors. MKo was taking main care of this case. MKi had an equal contribution to this case report. YK was supervising MKo and MKi. SG was performing PCI in this case. OK was investigating the novelty and the rarity of this case. SK was exploring the literature relating to this case. TM performed writing of the main part of this manuscript. KO had the initial concept of this manuscript preparation and provided this manuscript with deep insight. YM supervised collaboration of the cardiology team. All authors read and approved the final manuscript.

## Conflict of Interest

The authors declare that the research was conducted in the absence of any commercial or financial relationships that could be construed as a potential conflict of interest.
